# Vasoactivity and Vasoconstriction Changes in Cattle Related to Time off Toxic Endophyte-Infected Tall Fescue

**DOI:** 10.3390/toxins8100271

**Published:** 2016-09-22

**Authors:** James L. Klotz, Glen E. Aiken, Jessica R. Bussard, Andrew P. Foote, David L. Harmon, Ben M. Goff, F. Neal Schrick, James R. Strickland

**Affiliations:** 1 USDA-ARS, Forage-Animal Production Research Unit, Lexington, KY 40546, USA; glen.aiken@ars.usda.gov (G.E.A.); jrstric@clemson.edu (J.R.S.); 2 Department of Plant and Soil Sciences, University of Kentucky, Lexington, KY 40546, USA; jrb5218@gmail.com (J.R.B.); ben.goff@uky.edu (B.M.G.); 3 Department of Animal and Food Sciences, University of Kentucky, Lexington, KY 40546, USA; andrew.foote@ars.usda.gov (A.P.F.); dharmon@uky.edu (D.L.H.); 4 Department of Animal Science, University of Tennessee, Knoxville, TN 37996, USA; fschrick@utk.edu

**Keywords:** bovine, ergot alkaloids, tall fescue, vasoconstriction

## Abstract

Previous research has indicated that serotonergic and α-adrenergic receptors in peripheral vasculature are affected by exposure of cattle grazing toxic endophyte-infected (E+; *Epichlöe coenophialia*) tall fescue (*Lolium arundinaceum*). The objective of this experiment was to determine the period of time necessary for the vascular effects of ergot alkaloids to subside. Two experiments were conducted to investigate changes in vascular contractile response and vasoconstriction over time relative to removal from an ergot alkaloid-containing E+ tall fescue pasture. In Experiment 1, lateral saphenous vein biopsies were conducted on 21 predominantly Angus steers (357 ± 3 kg body weight) at 0 (*n* = 6), 7 (*n* = 6), 14 (*n* = 5), or 28 days (*n* = 4) after removal from grazing pasture (3.0 ha; endpoint ergovaline + ergovalinine = 1.35 mg/kg DM) for 126 days. In Experiment 2, lateral saphenous veins were biopsied from 24 Angus-cross steers (361 ± 4 kg body weight) at 0, 21, 42, and 63 days (*n* = 6 per time point) following removal from grazing tall fescue pastures (3.0 ha; first 88 days endpoint ergovaline + ergovalinine = 0.15 mg/kg DM; last 18 days endpoint ergovaline + ergovalinine = 0.57 mg/kg DM) for 106 total days. Six steers (370 ± 18 kg body weight) off of bermudagrass pasture for the same time interval were also biopsied on Day 0 and Day 63 (*n* = 3 per time point). Additionally, in Experiment 2, cross-sectional ultrasound scans of caudal artery at the fourth coccygeal vertebra were taken on Days 0, 8, 15, 21, 29, 36, 42, and 45 to determine mean artery luminal area to evaluate vasoconstriction. In both experiments, steers were removed from pasture and housed in a dry lot and fed a corn silage diet for the duration of biopsies and ultrasound scans. Biopsied vessels used to evaluate vasoactivity were cleaned, incubated in a multimyograph, and exposed to increasing concentrations of 4-Bromo-3,6-dimethoxybenzocyclobuten-1-yl) methylamine hydrobromide (TCB2; 5HT_2A_ agonist), guanfacine (GF; α_2A_-adrenergic agonist), and (*R*)-(+)-*m*-nitrobiphenyline oxalate (NBP; α_2C_-adrenergic agonist) in both experiments and ergovaline (ERV) and ergotamine (ERT) in Experiments 1 and 2, respectively. In Experiment 1, days off pasture × agonist concentration was not significant (*p* > 0.1) for all four compounds tested. In Experiment 2, GF, NBP, TCB2 and ERT were significant for days off pasture × agonist concentration interaction (*p* < 0.02) and vasoactivity increased over time. Vasoactivity to agonists was reduced (*p* < 0.05) when steers were initially removed from E+ tall fescue pasture compared to bermudagrass, but did not differ by Day 63 for any variable. Luminal areas of caudal arteries in steers grazed on E+ tall fescue relaxed and were similar to steers that had grazed bermudagrass for 36 days on non-toxic diet (*p* = 0.15). These data demonstrate changes in peripheral vasoactivity and recovery from vasoconstriction occur beyond five weeks off toxic pasture and 5HT_2A_ receptors appear to be more dramatically affected in the lateral saphenous vein by grazing E+ tall fescue pasture than adrenergic receptors.

## 1. Introduction

Ergot alkaloids produced by the endophyte *Epichloë coenophiala* that is found in tall fescue (*Lolium arundiaceum*) can alter cardiovascular function [1] and induce constriction in vascular tissue of extremities of animals grazing tall fescue [2]. Livestock consuming ergot alkaloids are less able to regulate body temperature, leading to a higher susceptibility to heat stress in warm air temperatures [3,4]. When cattle from tall fescue systems are transported to feedyards, physiological stressors from ergot alkaloid toxicosis can be combined with the stress of transport to increase the risk of morbidity and death loss [5]. An unknown factor in this interaction is the timeframe for vasculature to recover from ergot alkaloid exposure after cattle are removed from toxic endophyte-infected (E+) tall fescue pastures.

Previous vascular work has demonstrated a decreased vasoactivity associated with prior exposure to ergot alkaloids through grazing at the peripheral [6,7] and gastrointestinal vascular beds [8]. This suppressed vasoactivity was not evident in cattle that had been removed from an E+ tall fescue pasture treatment and finished on an ergot alkaloid-free diet for up to 103 days prior to slaughter [7]. Work that has evaluated how livestock recover from fescue toxicosis or ergot alkaloid exposure has shown recovery times that varied depending on the variable measured. Body temperatures of cattle removed from E+ tall fescue treatment normalized within a period of time that has ranged from seven days [9] up to 30 days [10], and is likely dependent on influence of the ambient temperature–humidity index. Measurements of urinary alkaloids in steers switched from a toxic E+ to endophyte-free tall fescue pasture decreased below detectable levels in less than four days [11]. Suppressed prolactin concentrations, long used as an indicator of ergot alkaloid exposure, have been shown to increase and stabilize in less than two weeks [10]. In the same study, however, Aiken et al. [10] reported that ergot alkaloid-induced vasoconstriction of the caudal artery was not alleviated after 30 days on a non-toxic diet. Based on these findings, it was hypothesized that time frames associated with measures such as serum prolactin concentrations, rectal temperatures, and urinary alkaloids that have been used to indicate livestock recovery from ergot alkaloid exposure do not encompass the time necessary for vascular recovery. The objective of these experiments was to measure the changes in vasoactivity and vasoconstriction in steers relative to removal from a toxic endophyte-infected tall fescue pasture.

## 2. Results

### 2.1. Animal Responses

**Figure 1:**
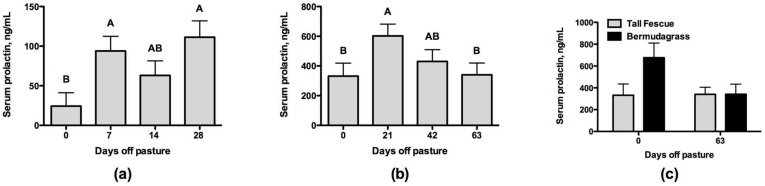
Serum prolactin concentrations on the day of the lateral saphenous vein biopsy relative to the number of days steers had been off of pasture consuming a non-toxic diet for: (**a**) Experiment 1 (effect of Day was *p* = 0.01); (**b**) Experiment 2 tall fescue steers (effect of Day was *p* = 0.09); and (**c**) Experiment 2 bermudagrass steers compared to tall fescue steers on Day 0 (effect of Pasture was *p* = 0.08) and Day 63 (effect of Pasture was *p* = 0.99). Bars not sharing the same superscripts are different (*p* < 0.05).

Following removal of steers from the toxic endophyte-infected tall fescue pasture, serum prolactin levels increased with the number of days off of pasture (*p* = 0.01) for Experiment 1 (Figure 1a). In this experiment, prolactin levels were the lowest in steers biopsied on day 0 (*p* < 0.05). The prolactin levels in Experiment 2 had a much higher background relative to Experiment 1, but this higher background was consistent across all samples collected regardless of treatment. The prolactin levels in Experiment 2 tended to differ by day (*p* = 0.09; Figure 1b). Prolactin went up 21 days after removal from pasture when compared to Day 0 (*p* < 0.05) in Experiment 2, but concentrations decreased on Days 42 and 63 and were not greater than Day 0. When compared to steers that were taken off of a bermudagrass pasture, the steers that had been grazing tall fescue tended to have lower serum prolactin on Day 0 (*p* = 0.08), but not by Day 63 (*p* = 0.99; Figure 1c).

The effect of time off pasture on the vascular dimensions (inside and outside diameters) for lateral saphenous veins biopsied in Experiment 1 and 2 are presented in Table 1. In Experiment 1, inside diameter was the greatest by Day 14 (*p* < 0.05). The outside diameter of the veins did have a tendency to differ with days off pasture, due primarily to the drop on Day 7 (*p* < 0.05). In Experiment 2, the inside diameter increased with days off pasture from Day 0 to Day 63 (*p* < 0.05), whereas the outside diameter did not differ (Table 1).

### 2.2. Vasoactivity

#### 2.2.1. Ergot Alkaloids

**Figure 2:**
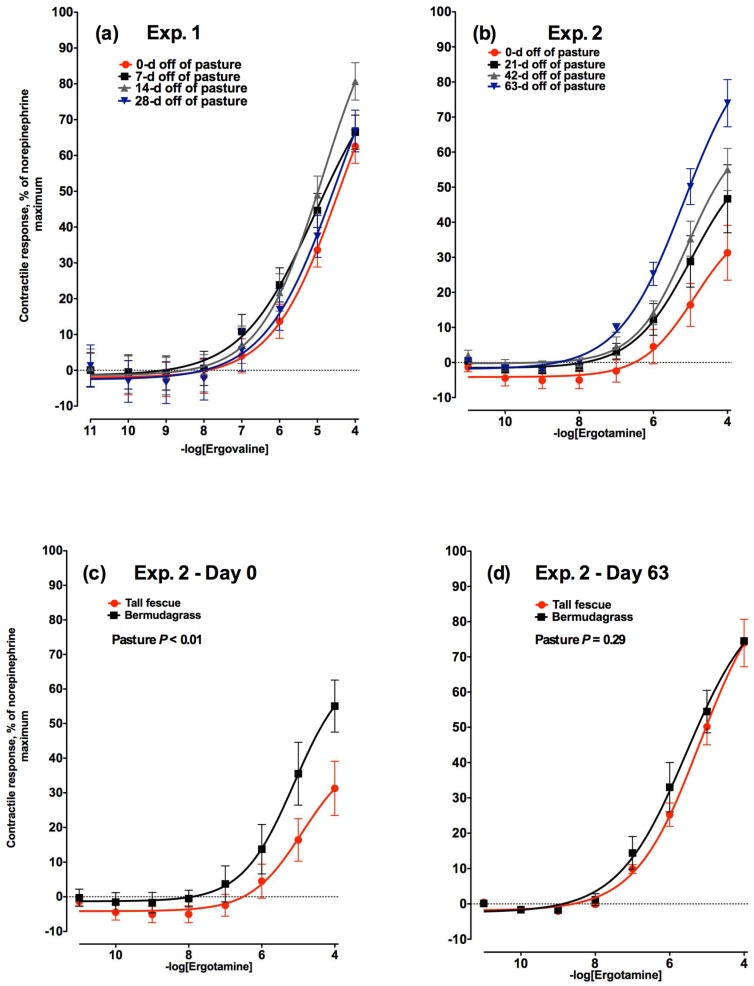
Contractile response of lateral saphenous veins (expressed as percentage of the maximal response induced by 1 × 10^−4^ M norepinephrine) to increasing concentrations of: (**a**) ergovaline in Experiment 1 at 0, 7, 14, and 28 days (−logEC_50_ = 4.46, 5.06, 4.85, and 4.50, respectively) after steers were removed from a tall fescue pasture and placed on a non-toxic diet (day × ergovaline concentration interaction was not significant (*p* = 0.66)); and (**b**) ergotamine in Experiment 2 at 0, 21, 42, and 63 days (−logEC_50_ = 4.89, 4.98, 5.09, and 5.18, respectively) after steers were removed from a tall fescue pasture and placed on a non-toxic diet (day × ergotamine concentration interaction was significant (*p* < 0.01)). Comparison of contractile responses of lateral saphenous veins biopsied from steers that had grazed toxic tall fescue (red lines) and non-toxic bermudagrass (black lines) pastures at (**c**) 0 and (**d**) 63 days after removal from pasture in Experiment 2.

The contractile response of the lateral saphenous vein to increasing concentrations of ergovaline did not differ across any of the four time points within 28 days evaluated in Experiment 1 (Figure 2a). All four time points had similarly shaped curves and the maximum vasoactivity to 1 × 10^−4^ M ergovaline addition was 63.8%, 70.5%, 83.8%, and 67.5% ± 5.8% for 0, 7, 14, and 28 days off of pasture, respectively.

In Experiment 2, the maximal contractile responses of bovine lateral saphenous veins biopsied on Days 0, 21, 42, and 63 to increasing concentrations of ergotamine were 31.3%, 46.7%, 55.1%, and 73.9% of the norepinephrine-induced maximum, respectively (at 1 × 10^−4^ M; Figure 2b). The onset of a contractile response was observed between 1 × 10^−6^ and 1 × 10^−8^ M ergotamine across all days (*p* < 0.05). Days 0 and 63 showed significant contractile responses between each other and compared to all other time points (*p* < 0.05). No difference was seen between contractile responses on Day 21 and Day 42 (*p* < 0.05). A concentration effect became significant at 1 × 10^−6^ M (*p* < 0.05). Effect of day × concentration also showed significant contractile responses at all time points (*p* < 0.05). Results demonstrated that lateral saphenous vein tissue was increasingly responsive to the ergot alkaloid, ergotamine, as time-off E+ tall fescue increased. There was no difference in the contractile response of lateral saphenous veins biopsied from bermudagrass steers to increasing concentrations of ergotamine on Day 0 (Figure 2c) or Day 63 (Figure 2d; *p* = 0.99).

#### 2.2.2. α_2A_-Adenergic Receptors

**Figure 3:**
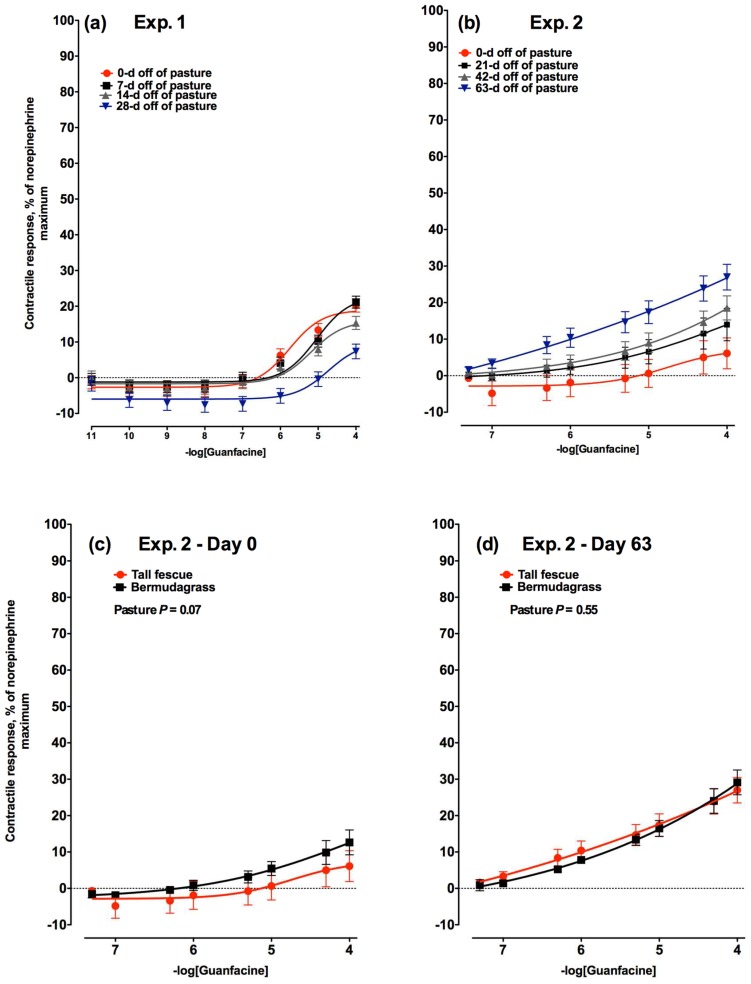
Contractile response of lateral saphenous veins (expressed as percentage of the maximal response induced by 1 × 10^−4^ M norepinephrine) to increasing concentrations of guanfacine HCl, an α_2A_-adrenergic receptor agonist in: (**a**) Experiment 1 on Days 0, 7, 14, and 28 (−logEC_50_ = 5.75, 5.05, 5.19, and 4.73, respectively) after steers were removed from a tall fescue pasture and placed on a non-toxic diet (day × guanfacine concentration interaction was significant (*p* < 0.01)); and (**b**) Experiment 2 on Days 0, 21, 42, and 63 (−logEC_50_ = 4.69, 4.91, 4.88, and 5.33, respectively) after steers were removed from a tall fescue pasture and placed on a non-toxic diet (day × guanfacine concentration interaction was significant (*p* < 0.01)). Comparison of contractile responses of lateral saphenous veins biopsied from steers that had grazed toxic tall fescue (red lines) and non-toxic bermudagrass (black lines) pastures at (**c**) 0 and (**d**) 63 days after removal from pasture in Experiment 2.

Lateral saphenous veins exposed to increasing concentrations of guanfacine HCl (GF), an agonist for α_2A_-adenergic receptors, had a significant interaction between GF concentration and time off of tall fescue in Experiment 1 (*p* < 0.01; Figure 3a). This was a consequence of a much lower response to GF by the veins biopsied from steers after 28 days off of pasture. The vascular response to GF in Experiment 2 also had a significant interaction between GF concentration and the time off of pasture (*p* < 0.01; Figure 3b). In this experiment, however, the contractile response to GF increased with number of days off of toxic tall fescue pasture. Days 0 and 21 did not differ, but the response on Day 42 was greater than on Day 0 (*p* < 0.05). α_2A_-Adenergic receptor vasoactivity was greatest by Day 63 (*p* < 0.05). There was no difference in the contractile response of lateral saphenous veins biopsied from bermudagrass steers to increasing concentrations of GF on Day 0 (Figure 3c) or Day 63 (Figure 3d; *p* = 0.96).

#### 2.2.3. α_2C_-Adrenergic Receptors

**Figure 4:**
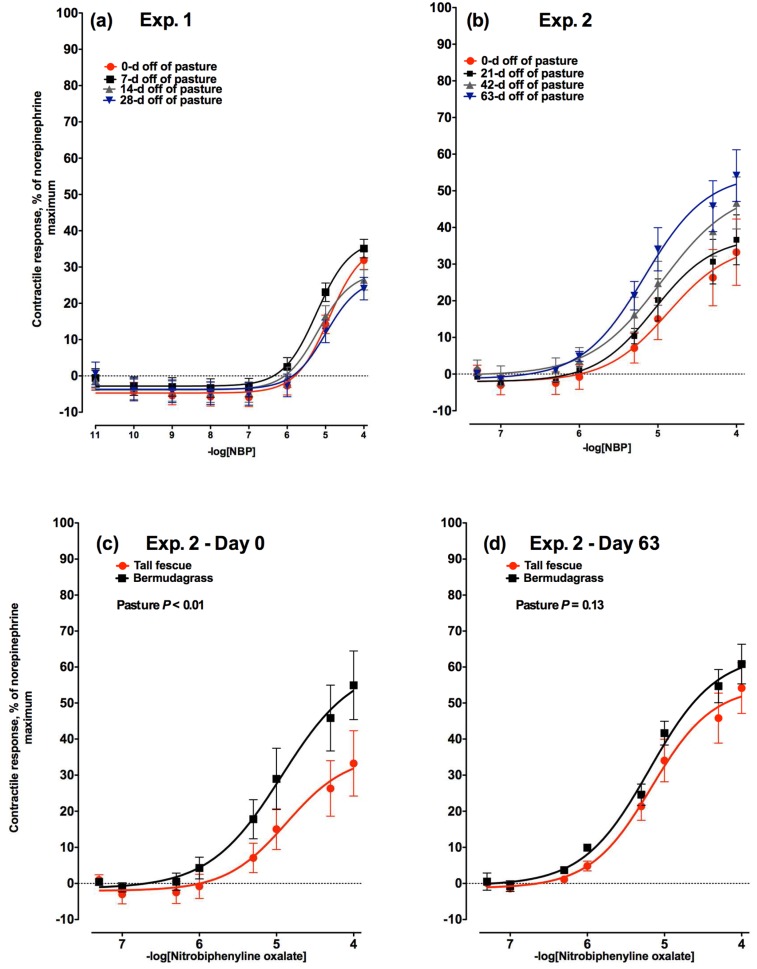
Contractile response of lateral saphenous veins (expressed as percentage of the maximal response induced by 1 × 10^−4^ M norepinephrine) to increasing concentrations of nitrobiphenyline oxalate (NBP), an α_2C_-adrenergic receptor agonist in: (**a**) Experiment 1 on Days 0, 7, 14, and 28 (−logEC_50_ = 4.91, 5.23, 5.19, and 4.99, respectively) after steers were removed from a tall fescue pasture and placed on a non-toxic diet (day × NBP concentration interaction was not significant (*p* = 0.41)); and (**b**) Experiment 2 on Days 0, 21, 42, and 63 (−logEC_50_ = 4.87, 5.05, 4.95, and 5.17, respectively) after steers were removed from a tall fescue pasture and placed on a non-toxic diet (day × NBP concentration interaction was significant (*p* = 0.017)). Comparison of contractile responses of lateral saphenous veins biopsied from steers that had grazed toxic tall fescue (red lines) and non-toxic bermudagrass (black lines) pastures at (**c**) 0 and (**d**) 63 days after removal from pasture in Experiment 2.

Increasing concentrations of nitrobiphenyline oxalate (NBP), an α_2C_-adrenergic receptor agonist, did not differ with respect to the number of days a steer was on a non-toxic diet (*p* = 0.41) in Experiment 1 (Figure 4a). The response to increasing concentrations of NBP in Experiment 2 (Figure 4b), began at the same concentration as Experiment 1 (1 × 10^−6^ M NBP), but, unlike Experiment 1, differed relative to the days off of pasture (*p* = 0.01). In agreement with absence of any time related differences in the 28-day time frame in Experiment 1, the contractile responses observed on the Day 0 and Day 21 biopsies did not differ (*p* > 0.05). The vasoactivity in response to stimulation of the α_2C_-adrenergic receptor with NBP increased with time and by Day 42 was different from Day 0 (*p* < 0.05), but not Day 21. Similarly, the level of vasoactivity increased to a maximum after 63 days off of tall fescue pasture when compared to Days 0 and 21 (*p* < 0.05), but the contractile response on Day 63 was not different from Day 42. There was no difference in the contractile responses of lateral saphenous veins biopsied from bermudagrass steers to increasing concentrations of NBP on Day 0 (Figure 4c) or Day 63 (Figure 4d; *p* = 0.73).

#### 2.2.4. 5-Hydroxytryptamine_2A_ Receptors

**Figure 5:**
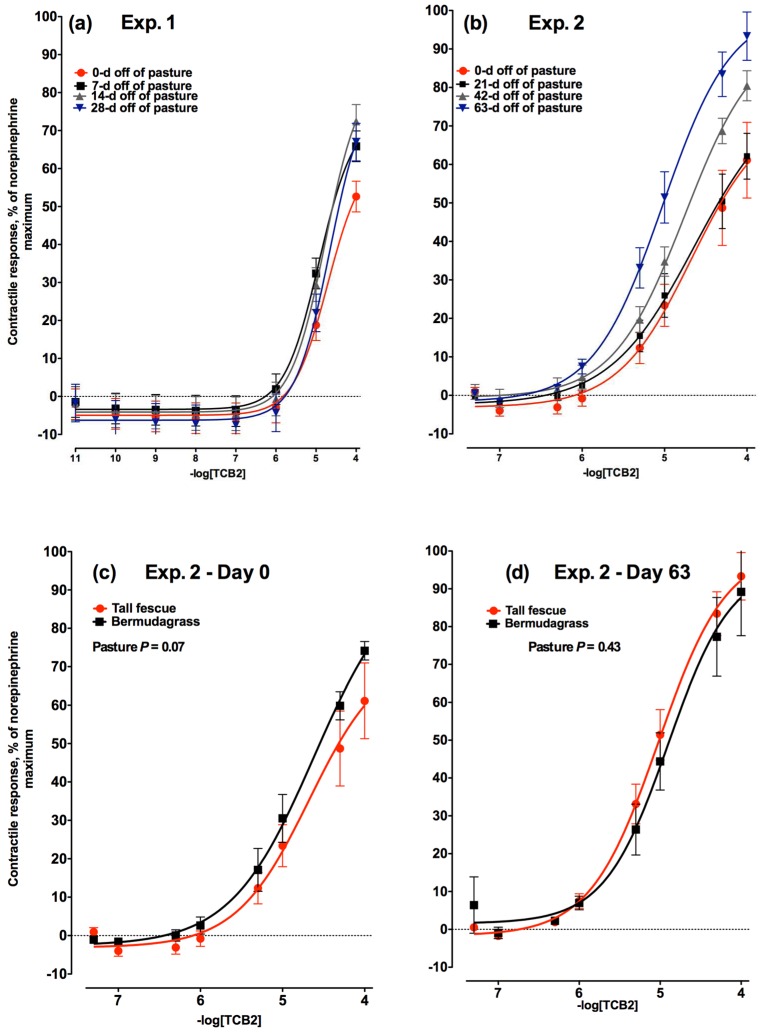
Contractile response of lateral saphenous veins (expressed as percentage of the maximal response induced by 1 × 10^−4^ M norepinephrine) to increasing concentrations of TCB2, an 5-hydroxytryptamine_2A_ receptor agonist in: (**a**) Experiment 1 on Days 0, 7, 14, and 28 (−logEC_50_ = 4.71, 4.92, 4.76, and 4.65, respectively) after steers were removed from a tall fescue pasture and placed on a non-toxic diet (day × TCB2 concentration interaction was not significant (*p* = 0.39)); and (**b**) Experiment 2 on Days 0, 21, 42, and 63 (−logEC_50_ = 4.67, 4.64, 4.75, and 5.02, respectively) after steers were removed from a tall fescue pasture and placed on a non-toxic diet (day × TCB2 concentration interaction was significant (*p* < 0.01)). Comparison of contractile responses of lateral saphenous veins biopsied from steers that had grazed toxic tall fescue (red lines) and non-toxic bermudagrass (black lines) pastures at (**c**) 0 and (**d**) 63 days after removal from pasture in Experiment 2.

The contractile response to increasing concentrations of TCB2, an agonist of 5-hydroxytyrptamine_2A_ (also known as serotonin; 5HT) receptors did not change (*p* = 0.39) relative to the number of days a steer was off of tall fescue in Experiment 1 (Figure 5a). In both Experiment 1 and 2 (Figure 5b), the concentration of TCB2 where contractile responses were initially observed was 1 × 10^−6^ M. In Experiment 2, after 21 days off of tall fescue there was no change in the vasoactivity of the 5HT_2A_ receptor supporting the observations in Experiment 1. However, the vasoactivity increased by Day 42 (*p* < 0.05) compared to Days 0 and 21 and increased again in intensity by Day 63 (*p* < 0.05). There was no difference in the contractile response of lateral saphenous veins biopsied from bermudagrass steers to increasing concentrations of TCB2 on Day 0 (Figure 5c) or Day 63 (Figure 5d; *p* = 0.53).

#### 2.2.5. Comparison to Non-Toxic Control Steers

To evaluate if the changes in vasoactivity were due to ergot alkaloid exposure and not changes due to time or environment, vasoactivity was evaluated for all agonists in a non-toxic bermudagrass control group and compared to the vasoactivity of veins biopsied from tall fescue steers at the beginning (Day 0) and end (Day 63) of Experiment 2. For all three receptor agonists, the vasoactivity was lower (NBP; *p* < 0.01; Figure 4c) or tended to be lower (TCB2 and GF; *p* = 0.07; Figure 5c and Figure 3c, respectively) in lateral saphenous veins biopsied from steers on toxic tall fescue after 0 days off of pasture when compared to steers from bermudagrass pastures. Conversely, following 63 days of consuming an ergot alkaloid-free diet, there were no longer any differences in vasoactivity between steers that had formerly grazed tall fescue or bermudagrass at any of the receptors selectively targeted with agonists (*p* > 0.13; Figure 3d, Figure 4d, and Figure 5d).

Comparison of contractile responses of saphenous vein from tall fescue steers to that of bermudagrass steers on Day 0 yielded maximal responses to ergotamine of 31.3% and 55.1%, respectively. Steers at 0 days off tall fescue pasture had a much lower (*p* < 0.05) contractile response to ergotamine compared to veins biopsied from steers 0 days off bermudagrass pasture (Figure 2c). However, by Day 63 contractile responses of tall fescue steers were not different from those of bermudagrass steers resulting in maximal contractile responses of 73.9% for tall fescue steers and 74.5% for bermudagrass steers (Figure 2d).

### 2.3. Vasoconstriction

**Figure 6:**
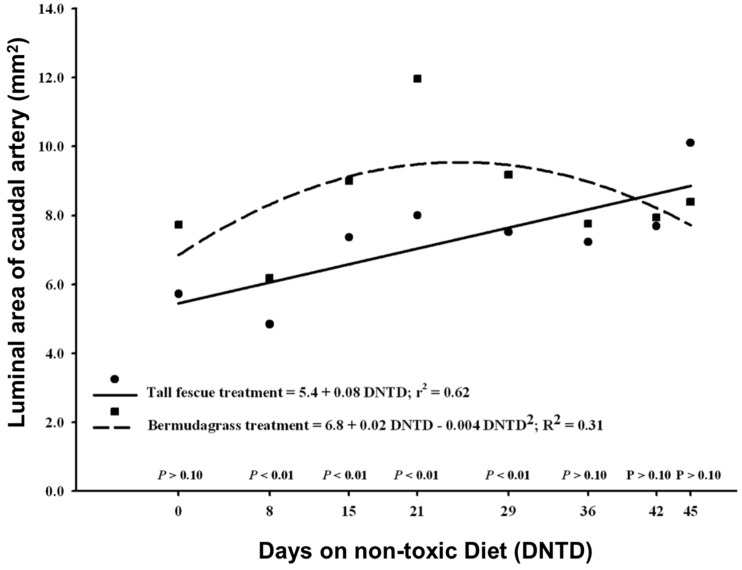
Doppler ultrasound measures of the change in luminal area of caudal arteries of steers that had been removed from either a toxic tall fescue or a non-toxic bermudagrass pasture. Luminal areas were regressed over days on a non-toxic diet relative to removal from pasture.

Luminal areas of the caudal artery of steers previously grazed on toxic tall fescue increased linearly over time (Figure 6). A curvilinear increase was found for luminal areas of steers that had previously grazed bermudagrass (Figure 6). At Day 0 luminal areas showed no significant differences (*p* = 0.297) of least squares means between tall fescue and bermudagrass treatments. By Day 7, significant differences were observed in lumen areas between tall fescue and bermudagrass steers (*p* = 0.003). Tall fescue steers exhibited lower lumen areas compared to bermudagrass steers. Lumen areas of the caudal artery in tall fescue steers relaxed and were similar to steers that had grazed bermudagrass by Day 36 on the non-toxic diet (*p* > 0.10).

## 3. Discussion

Experiments 1 and 2 were designed to evaluate vascular recovery of beef steers following an exposure to ergovaline while grazing of toxic endophyte-infected tall fescue. The steers used in Experiments 1 and 2 were exposed to differing levels of ergovaline in the pasture for differing lengths of time. While this was a consequence of environmental and experimental design factors in two independent grazing studies [12,13] conducted prior to the present study, the steers biopsied in both experiments had sufficient levels of exposure to cause ergot alkaloid toxicity leading into these experiments. Prior work has demonstrated the onset of a vasoconstrictive effect to dietary ergot alkaloid exposure is fairly rapid. A study that fed beef heifers ergovaline at 0.39 and 0.79 mg of ergovaline/kg of DM demonstrated a vasoconstrictive response in the caudal artery at 51 and 27 h following initiation of the treatment [14]. This was an ergovaline level (0.39 mg/kg) that was below what was measured in the pasture in Experiment 2, but was still capable of generating a measurable negative response.

In addition to vasoconstriction, a lowered serum prolactin concentration is another hallmark of ergot alkaloid exposure in cattle. Concentrations of serum prolactin in cattle can also be affected by photoperiod [15], acute changes in ambient temperature [16], and various forms of stress [17,18]. Consequently, there is a wide range in reported values across individuals as well as studies. Examples of studies that have measured serum prolactin concentrations in cattle on pasture have reported levels of 36.0 [19], 50 [20], 108 [21], and 194 ng/mL [22] for control or endophyte-free pasture treatments. Conversely, for these same studies prolactin concentrations measured in steers on the ergot alkaloid-containing pasture treatment were 3.6 [19], 16 [20], 17 [21], and 82 ng/mL [22]. It is important to note that this wide range in reported concentrations makes comparisons across studies tenuous, due to the numerous factors that can influence the measurement of prolactin at a single point in time. Rather, the trends within a study relative to treatments are more germane in the establishment of an effect.

Steers used in Experiment 1 demonstrated a suppressed concentration of serum prolactin on Day 0 that recovered after seven days on a non-toxic diet. This is indicative of ergot alkaloid exposure and is similar to timelines reported by Aiken et al. [10] where prolactin returned to levels not different from the non-toxic control by 10 to 15 days on a non-toxic diet. The prolactin data for Experiment 2 did not follow a trend similar to Experiment 1, as all data were unusually elevated. In addition to reanalysis of some samples to confirm the observed results, Experiment 2 was conducted at the same time of year as Experiment 1 ruling out an effect of photoperiod on prolactin concentrations. The levels of stress that steers underwent were not different across experiments and there were no extreme differences in weather across the two years. Looking at the data within Experiment 2, the Day 0 prolactin concentrations were higher than expected; however, relative to the levels after 21 days on a non-toxic diet, they were lower. Furthermore, the Day 0 prolactin levels were also lower in steers coming off of toxic endophyte-infected tall fescue pastures compared to steers coming off of bermudagrass pastures. This combined with the vasoconstriction observed in the caudal artery of steers that had grazed the toxic endophyte-infected tall fescue pastures indicates that these steers had adequate exposure to ergot alkaloids.

### 3.1. Vasoactivity of the Lateral Saphenous Vein

Exposure to ergot alkaloids, specifically ergopeptine alkaloids like ergovaline or ergotamine has been shown to cause a significant decrease in subsequent vasoactivity. Specifically, this has been demonstrated using a bovine mesenteric artery and vein bioassay with both chronic ruminal dosing [8] and acute direct incubation [23] with ergovaline, which resulted in large decreases in vasoactivity after exposure. Prior work that evaluated the vasoactivity of the lateral saphenous vein in relation to cattle that had grazed toxic endophyte-infected or endophyte-free tall fescue demonstrated that veins from the toxic E+ were not as responsive [6,7]. Klotz et al. [7] went on to demonstrate that vascular alterations induced by fescue toxicosis were ameliorated in steers following a 91 to 103 day finishing period. This phenomenon of reduced or suppressed vasoactivity was present in the current study, but was only evident in Experiment 2 where the timeline was long enough to permit an observed increase from Day 0 or 21. Additionally, the vascular recovery appeared to have been reached by Day 63 as the vasoactivity of the veins biopsied from the toxic tall fescue steers no longer differed from the non-toxic bermudagrass control steers.

The observed changes in vasoactivity could be related to structural changes such as a hyperplastic or hypertrophic response to alkaloid exposure. Vascular thickening has been reported in previous work that associated ergot alkaloid exposure with a decrease in luminal diameter [24]. The vascular dimension data reported for Experiment 2 clearly demonstrated a decreasing wall thickness associated with an increasing time off pasture. This dimensional change exceeded the time for observed changes in prolactin concentrations in the current study that are typically associated with recovery from fescue toxicosis. Ergonovine, ergocryptine, and ergovaline have all been shown to increase vascular smooth muscle cell growth in vitro, and this would suggest a potential for the thickening of the tunica media smooth muscle layer [25].

All three receptor types evaluated in the current study were affected by consumption of ergot alkaloid-containing forage. The alteration in receptor-stimulated response is a result of the ergot alkaloid interference with the function of the vascular smooth muscle. The function of a 5HT_2A_ receptor is associated with thermoregulation and vasoconstriction [26]. Prior work has demonstrated that ergopeptine alkaloids, like ergovaline and ergotamine, interact with this receptor [7,27]. Other in vitro studies have demonstrated a sustained contractile response following exposure to ergopeptine alkaloids that does not diminish over time [27,28,29]. The prolonged stimulation of 5HT_2A_ receptors has been shown to result in a phosphorylation of associated G-proteins and an attenuation of signal transduction [30]. This possible alteration in 5HT_2A_ receptor signaling activity is the likely cause behind decreased contractile response when this receptor was selectively targeted with the agonist TCB2 in the current study. As the receptor population negatively affected by the ergot alkaloids consumed on pasture turns over and is replaced with ergot alkaloid-naïve receptors, the vasoactivity of the veins is increased. Looking at the response to TCB2 in Experiments 1 and 2, this occurred after steers had been on a non-toxic diet for over four weeks.

The adrenergic receptors were also selected for analysis in the current study because of their involvement in vasoconstriction in veins [31]. The α_2_-adrenergic receptors have been reported to have a generally higher affinity for ergopeptines compared to the α_1_-adrenergic receptors [32]. Additionally, the vasoactivity of this α_2_-subtype of receptor has also been shown to be affected by exposure to ergot alkaloids, whereas the α_1_-adrenergic receptor was not [33]. Additionally, ergotamine (and presumably ergovaline) has been shown to act as a partial agonist and competitive antagonist on α-adrenergic receptors [34]. The agonist NBP was more vasoactive than GF in the bovine lateral saphenous vein in both experiments. In addition, in both Experiment 1 and 2, the antagonistic effect of exposure to ergopeptine alkaloids (ergovaline) on both the adrenergic receptors evaluated appeared to last beyond four weeks after cattle were placed on a non-toxic diet. This indicates that adrenergic receptors are negatively affected by ergot alkaloid exposure, regardless of the pharmacologic subtype.

The results of the present study support those of Klotz et al. [7] where decreases in vasoactivity were reversed by extended time off of an ergot alkaloid-containing diet. The current study provides further evidence that ergot alkaloid-induced vascular changes in cattle are reversible and that this appears to require a minimum of 35 days free of ergot alkaloids (when considering the vasoconstriction and vasoactivity data). While data for ergot alkaloid concentrations recovered from vascular tissue were not obtained in this study, in vitro bioaccumulation of ergot alkaloids [35,36] and persistence of these compounds in inducing contractile response [27,28,29,37,38] have been shown in previous studies. In vivo studies have also suggested that ergot alkaloid accumulation can occur. Zbib et al. [39] reported trace amounts of ergovaline in kidney, liver, and abdominal fat samples taken from ewes fed a perennial ryegrass diet containing ergovaline. There is also some evidence that ergot alkaloids may accumulate in bovine subcutaneous adipose [40]. This potential for ergot alkaloid build-up in tissues of exposed animals may be an explanation behind the delay in vascular recovery. As was hypothesized in the current study, contractile responses of lateral saphenous veins biopsied from tall fescue steers were much lower compared to those of control steers that had previously grazed bermudagrass pasture on 0 day off pasture. Chronic alkaloid exposure over time affects the function of adrenergic and serotonergic receptors [6,7,33]. Once the steers were removed from E+ tall fescue pastures, dissipation of ergot alkaloids from cellular binding sites on receptors or a gradual turnover of alkaloid-receptor complexes may lead to increased contractility of vascular tissues as seen on Day 63 with saphenous veins from tall fescue steers exhibiting contractile responses similar to those of bermudagrass steers. It is possible that whether an ergopeptine alkaloid like ergovaline is bound to receptor associated with smooth muscle, or in a storage tissue like adipose that it could result a gradual release or turnover. This gradual release of ergopeptine alkaloids from a receptor could result in rebinding of alkaloids. This could delay total recovery long after cessation of alkaloid intake has occurred and might explain why reported recovery exceeds likely receptor turnover rates.

### 3.2. Vasoconstriction of the Caudal Artery

Color Doppler ultrasonography has the repeatability to provide objective in vivo measures of vasoconstriction in cattle induced by ergot alkaloids comparable to that of multimyograph experiments. In addition, the non-invasive manner of Doppler ultrasonography allows for practical application of this method in field research, lessening both stresses on livestock, labor, and resources needed to study vascular changes in peripheral tissues of the animal.

The absence of any difference between the luminal areas of tall fescue and bermudagrass steers on Day 0 may have been due to the stress of placement into an unfamiliar environment when steers were taken off of pasture and placed into pens. Additionally, it has been previously noted that the greater variation in caudal artery lumen areas seen in cattle on non-toxic diets with an unabated ability to respond to environmental conditions [41] when compared to toxic tall fescue counterparts could have also contributed. Initial vasoconstriction seen in steers that had previously grazed E+ tall fescue was consistent with that seen in heifers fed E+ tall fescue seed in pen studies by Aiken et al. [14,41]. The relaxation of caudal arteries of tall fescue steers over time and the similarity to lumen areas of bermudagrass steers by Day 35 in the current study agrees with detection of the increasing contractile responses of saphenous veins of tall fescue steers and similarity of saphenous veins of bermudagrass steers seen in the multimyograph studies evaluating vasoactivity. These combined results provide strong evidence that these signs are indicative of vascular changes seen in cattle recovering from fescue toxicosis. These results show that time to recovery extends past the 10 to 14 days previously determined for prolactin concentrations by Aiken et al. [10].

## 4. Conclusions

Multimyograph studies provide an effective method of testing direct effects of specific compounds on vascular tissue in a laboratory setting; however this type of direct exposure may not replicate the true physiological response in the whole animal. Doppler ultrasonography is an accurate tool for determination of real-time vascular sensitivities and responses to broad application of toxicants such as ergot alkaloids. Through the use of these very different in vitro and in vivo techniques that evaluated vasoactivity and vasoconstriction, respectively, the vascular recovery following exposure to ergot alkaloid-containing pasture has been more clearly defined. Results from both techniques indicate that vascular recovery had still not occurred by Day 28. Accordingly, steers should be removed from toxic endophyte-infected fescue pastures for a minimum 35 to 42 days to obtain vascular recovery from ergot alkaloid exposure that will facilitate an increase in vasoactivity, a reduction in vasoconstriction, and a corresponding decreased susceptibility to heat stress. Because actual clearance of ergot alkaloids was not determined in this study, there is a need for further research in this area. Furthermore, recovery from ergot alkaloid exposure could take longer for cattle with greater bioaccumulation of ergot alkaloids in the vasculature than the steers used herein.

## 5. Materials and Methods

### 5.1. Animal Management

All experimental procedures involving live steers were approved by the University of Kentucky Institutional Animal Care and Use Committee (# 2011-0791) and conducted at the University of Kentucky C. Oran Little Research Center in Woodford County.

#### 5.1.1. Experiment 1

Twenty-four predominantly Angus steers (357 ± 3 kg body weight) grazed E+ Kentucky 31 tall fescue pastures (3 ha) for 126 days during a separate grazing study [12]. The total ergovaline (ergovaline + ergovalinine) concentration of the tall fescue pastures just prior to removal of the steers was 1.35 mg/kg DM. To accommodate venous biopsies, steers were moved from pasture in two groups of 12 spaced two days apart and housed in a dry lot with a concrete floor and partially covered in a three-sided barn. From this point forward steers were bunk-fed corn silage and with soybean hull mixed diet. Steers had ad libitum access to water and mineral supplement.

#### 5.1.2. Experiment 2

Twenty-four predominately Angus steers (361 ± 4 kg body weight) grazed E+ Kentucky 31 tall fescue pastures for 88 days during a separate grazing study [13]. In this study [13], it was determined at its conclusion that the tall fescue pastures were minimally infected and had low levels of total ergovaline (ergovaline + ergovalinine = 0.15 ± 0.50 mg/kg DM). In response to this finding, the steers that were to biopsied in the current study were moved to alternative 3 ha tall fescue pastures that had higher infection levels (ergovaline + ergovalinine = 0.57 mg/kg DM) for an additional 18 days prior to moving to the dry lot for Day 0 biopsies. Six mixed breed steers (370 ± 18 kg) that had grazed bermudagrass (*Cynodon dactylon* (L.) Pers.) pasture (3.0 ha) during the same time interval were used as non-toxic controls. Steers were removed from the Kentucky 31 pasture in two groups of 12 spaced two days apart. Six steers were removed from bermudagrass pasture on the day between the two days that the two groups of steers were removed from E+ tall fescue. All steers were housed in the same facility and fed the same non-toxic diet described in Experiment 1.

### 5.2. Lateral Saphenous Vein Biopsy

#### 5.2.1. Experiment 1

Cranial branches of lateral saphenous veins were biopsied in the months of August and September and processed as described in detail by Klotz et al. [42]. Briefly, a steer was restrained in a left lateral recumbency using a tilt table (Spring-O-Matic Inc., Marion, KS, USA). Hair was removed and the biopsy site was cleaned with povidone-iodine soap and disinfected with 70% ethanol. The surgical site was locally anesthetized with a line block with 2% lidocaine (2% injectable; The Butler Co., Dublin, OH, USA) that was applied proximal to the incision line. Once the vessel was identified, ligatures were placed, the isolated section (~3 cm) of vein was removed, and the skin was sutured. Isolated venous tissue was placed in a modified Krebs-Henseleit oxygenated buffer solution (95% O_2_/5% CO_2_; pH = 7.4; mM composition = d-glucose, 11.1; MgSO_4_, 1.2; KH_2_PO_4_, 1.2; KCl, 4.7; NaCl, 118.1; CaCl_2_, 3.4; and NaHCO_3_, 24.9; Sigma Chemical Co., St. Louis, MO, USA) for transport, and kept on ice until processed. Immediately after the biopsy surgery, steers received penicillin (Procaine G, 6600 U/kg of BW; Norbrook Inc., Kansas City, MO, USA) and flunixin meglumine (Flunixiject, 1.1 mg/kg of BW; IVX Animal Health Inc., St. Joseph, MO, USA) and were returned to the pens. Administration of flunixin meglumine was continued for three days postoperatively.

Biopsies occurred 0 (*n* = 6), 7 (*n* = 6), 14 (*n* = 5), and 28 (*n* = 4) days after removal from pasture (three biopsies were not completed due to health and behavior complications). A maximum of three steers were biopsied on a given day relative to their removal from pasture and all associated myograph experiments (described below) were run the same day as the biopsy.

#### 5.2.2. Experiment 2

Biopsies of the cranial branch of lateral saphenous veins were conducted in the months of August through October as described for Experiment 1. Steers from the E+ tall fescue pasture (*n* = 6 per time point) were biopsied 0, 21, 42, and 63 days after their removal from pasture. Steers from the non-toxic bermudagrass pastures (*n* = 3 per time point) were biopsied 0 and 63 days after their removal from pasture. A maximum of three steers were biopsied on a given day relative to removal from pasture.

### 5.3. Myograph—Vasoactivity

Biopsied blood vessels from both experiments were used in vitro to evaluate changes in contractile response to increasing concentrations of adrenergic and serotonergic agonists and ergot alkaloids. Contractile response experiments were run the same day as biopsy. Tissue processing for all vein segments followed methods validated by Klotz et al. [43]. Tissue preparation for experiments consisted of removal of excess fat and connective tissue. Cleaned segments were sliced into 2- to 3-mm cross sections. Cross-sections were examined under a dissecting microscope (Stemi 2000-C, Carl Zeiss Inc., Oberkochen, Germany) at 12.5× magnification to measure dimensions for assurance of consistent segment size and to verify physical integrity of tissue. Cross-sections were suspended horizontally in a 5-mL tissue bath (DMT610M Multichamber myograph, Danish Myo Technologies, Atlanta, GA, USA) containing continuously oxygenated modified-Krebs Henseleit buffer (95% O_2_/5% CO_2_; pH = 7.4; 37 °C), with 3 × 10^−5^ M desipramine and 1 × 10^−6^ M propranolol (Sigma Chemical Co.) to inactivate catecholamine-neuronal uptake and β-adrenergic receptors, respectively. After equilibration to 1 g of tension (~1.5 h), tissues were exposed to the α-adrenergic agonist norepinephrine (1 × 10^−4^ M; Sigma Chemical Co.) to verify tissue viability and as a reference for normalization of the corresponding contractile responses.

Cross-sections of lateral saphenous veins were run in duplicate from each steer for each contractile response treatment. After recovery from the 1 × 10^−4^ M norepinephrine addition (45 to 60 min) and the reestablishment of the 1-g baseline tension, alkaloid additions occurred in 15-min intervals. Each 15-min interval consisted of a 9-min treatment incubation period, followed by a washout period during which duplicate aliquots of buffer minus the treatment were incubated with the vein segment for two 2.5-min periods, followed by a final buffer replacement and 1-min incubation before the next addition.

For Experiment 1, the treatment additions were an ergot alkaloid ergovaline tartrate (93% purity; supplied by Forrest T. Smith, Auburn University, Auburn, AL, USA), a serotonin (5HT) receptor agonist selective for the 5HT_2A_ receptor (4-bromo-3,6-dimethoxybenzocyclobuten-1-yl)methylamine HCl (TCB2; Tocris Biosciences, Ellisville, MO, USA), a selective α_2A_-adrenergic receptor agonist guanfacine HCl (GF; Tocris Biosciences), and a α_2C_-adrenergic receptor agonist (*R*)-(+)-*m*-nitrobiphenyline oxalate (NBP; Tocris Biosciences). The contractile response curves were constructed with 8 consecutive additions every 15 min at fixed concentrations ranging from 1 × 10^−11^ to 1 × 10^−4^ M in the tissue bath. For Experiment 2, the ergot alkaloid ergotamine tartrate (≥97% purity, Fluka, as distributed by Sigma Chemical Co.) was used in the same dilutions as Experiment 1, because of a shortage of purified ergovaline. Ergotamine was selected because Klotz et al. [28] demonstrated that ergovaline and ergotamine produce similar contractile responses in the bovine lateral saphenous vein bioassay. The receptor agonists TCB2, GF, and NBP were used as in Experiment 1, however the range of concentrations tested were modified to 5 × 10^−8^ to 1 × 10^−4^ M to better characterize the response curve.

In both experiments, isometric contractions of lateral saphenous vein were recorded as grams of tension in response to exposure to norepinephrine and the myograph treatments. The data were digitally recorded using a Powerlab/8sp (ADInstruments, Colorado Springs, CO, USA) and Chart software (Version 7.2, ADInstruments). Contractile response was recorded as the greatest contractile response, in grams, within the 9-minute incubation after a treatment addition and corrected by the baseline tension recorded just before the addition of 1 × 10^−4^ M norepinephrine. Response data were normalized as a percentage of the maximal contraction produced by norepinephrine (1 × 10^−4^ M). Normalization compensated for variation of the tissue responsiveness due to differences in tissue size across individual cattle. To construct the concentration-response curves, the normalized data were plotted with GraphPad Prism (version 5.0f; San Diego, CA, USA). This graphical presentation used a nonlinear regression (sigmoidal concentration-response curve) to fit a line to contractile response data points for a treatment using the four-parameter equation:

(1) $$y=bottom+\;\frac{\left(top-bottom\right)}{\left\{1+10^{\left[\left(logEC_{50}-x\right)Hill\;slope\right]}\right\}},$$

where the top and bottom are plateaus in the units of the y-axis and the Hill slope is the numerical determination of the steepness of the curve. This calculation also allowed for the calculation of a compound’s potency in the bovine lateral saphenous vein bioassay expressed as the concentration the compound required to produce 50% of the observed contractile response (EC_50_).

### 5.4. Doppler Ultrasonography—Vasoconstriction

Steers in the last group to be biopsied on Day 63 (*n* = 6) and the non-toxic control steers (*n* = 6) from the bermudagrass pasture were used to monitor the level of vasoconstriction of the caudal artery using color Doppler ultrasonography. Ultrasound scanning sessions began at 1:00 p.m. and ended at approximately 2:00 p.m.. Ultrasound measures were taken during the experimental period at 0, 8, 15, 21, 29, 36, 42, and 45 days after being placed in the pens and fed the nontoxic diet. Day 0 was the day that steers were placed in the pens and therefore represents baseline measures. Ultrasound scans of the caudal artery at the 4th coccygeal (Cd4) vertebrae were measured using an Aloka 3500 Ultrasound Unit (Aloka, Inc., Wallingford, CT, USA) with a UST-5542 (13 MHz) linear array transducer set to a 2-cm depth. Five B-mode scans were taken at a frequency of 6.0 MHz to determine cross-sectional area of the caudal artery. Following freezing of an individual scan, frames stored in the cine memory of the unit were searched to store the image exhibiting the maximum flow signal and assumed to be at peak systolic phase. This flow signal was traced to estimate lumen area as validated by Aiken et al. [44].

### 5.5. Sample Collection and Analyses

Tall fescue tiller samples were collected from the holding pastures that the steers were held in during the interval between the conclusion of the separate grazing study and the initiation of the recovery studies. Samples were collected on Day 1 relative to the steers’ removal from pasture for both experiments. Single tillers were clipped at the crown from 25 randomly selected tall fescue plants. These samples were dried in a Botanique freeze drier (Model 18DX485A; Botanique Preservation Co., Peoria, AZ, USA) and ground to pass through a 1-mm screen (Cyclotec 1093 Sample Mill, Foss North America, Eden Prairie, MN, USA). Dried and ground tiller samples were composited and analyzed for ergovaline and ergovalinine content with high performance liquid chromatography and fluorescence detection using the procedure of Yates and Powell [45] as modified by Carter et al. [46].

Jugular blood samples were collected from each steer during the lateral saphenous vein biopsy for both Experiment 1 and 2. Samples were stored on ice. Serum was collected following centrifugation at 3000× g for 10 min at 20 °C. Using the procedures of Bernard et al. [47], serum prolactin analysis was conducted by the laboratory of F. N. Schrick (University of Tennessee, Knoxville, TN, USA). Many samples collected in Experiment 2 were rerun to confirm elevated prolactin concentrations that were observed across all treatments. The intra- and inter-assay CV for Experiment 1 were 7.03% and 7.89%, respectively, and the CV for Experiment 2 5.97% and 9.68%, respectively.

### 5.6. Statistical Analyses

#### 5.6.1. Myograph Data

All statistics were conducted using the Mixed models of SAS (SAS 9.3; SAS Inst. Inc., Cary, NC, USA) and for tests of fixed effects, the Satterthwaite approximation of denominator degrees of freedom was used. Contractile response data to receptor agonist and ergot alkaloid treatments were analyzed as a completely randomized design with a split-plot treatment design. The whole plot experimental unit was steer with the days off of pasture as the treatment factor. The biopsied blood vessel was the subplot experimental unit and the agonist concentration was the subplot treatment factor. The model included fixed effects of days off of pasture, agonist concentration, and the interaction. In Experiment 1, the contractile response data for TCB2 were log-transformed due to heterogeneous variance distribution. This transformation yielded normal distribution according to the Sharpiro–Wilk test (*W* = 0.97). In Experiment 2, contractile response data collected from steers that had been on the bermudagrass pasture were compared to data collected from steers that had been on E+ tall fescue for each agonist or ergotamine on Day 0 and 63. These analyses were conducted separately and used the same split-plot treatment design as above, but pasture replaced days off of pasture and the treatment factor for the whole plot. For the variables prolactin and vessel dimensions (inside and outside diameter), analysis of variance was conducted for the main effect of days off pasture.

For all statistical analyses, pairwise comparisons of least squares means (±SEM) were conducted only if the probability of a greater *F*-statistic in the ANOVA was significant for the tested effect. Means separation was conducted using least significant difference (LSD) feature in SAS and comparisons were considered significant at *p* ≤ 0.05, unless reported otherwise.

#### 5.6.2. Doppler Ultrasonography Data

Caudal artery luminal area data collected in Experiment 2 were analyzed using mixed models of SAS. Steer was considered the experimental unit. The days in pen (relative to removal from pasture) was analyzed as a continuous variable (linear and quadratic regression coefficients), and pasture treatment (E+ tall fescue versus bermudagrass) as a discrete variable [48]. Models containing only interactions between treatments and regression coefficients were analyzed to determine significant (*p* ≤ 0.10) linear and quadratic regression coefficients for each pasture treatment [49]. In the instance of a pasture treatment × days on non-toxic diet interaction (*p* ≤ 0.10), least square means for treatments were compared at seven-day intervals using the PDIFF option of SAS.

## Figures and Tables

**Table 1:** Inside and outside diameters of lateral saphenous veins biopsied on Days 0, 7, 14, and 28 for Experiment 1 and on Days 0, 21, 42, and 63 for Experiment 2.

Variable ^1^	Days on Non-Toxic Diet				SEM	*p*-Value ^2^
Experiment 1	**0**	**7**	**14**	**28**	-	-
i.d., mm	0.69 ^bc^	0.74 ^b^	1.00 ^a^	0.63 ^c^	0.04	<0.01
o.d., mm	2.72	2.57	2.82	2.81	0.08	0.07
Experiment 2	**0**	**21**	**42**	**63**	-	-
i.d., mm	0.71 ^c^	0.85 ^b^	0.89 ^b^	1.02 ^a^	0.03	<0.01
o.d., mm	2.90	2.94	2.92	2.85	0.04	0.50

^1^ i.d. = inside diameter and o.d. = outside diameter; ^2^ Probability of a greater F-statistic for the effect of days off of pasture. Significant at *p* < 0.05; ^abc^ Means within a row not containing like superscripts are different (*p* < 0.05).

## Abbreviations

The following abbreviations are used in this manuscript:

TCB2	4-Bromo-3,6-dimethoxybenzocyclobuten-1-yl)methylamine hydrobromide
GF	Guanfacine HCl
NBP	(*R*)-(+)-*m*-Nitrobiphenyline oxalate
ERT	Ergotamine tartrate
ERV	Ergovaline
E+	Endophyte-infected
5HT	5-hydroxytryptamine or serotonin
